# Commentary: Stereotactic Body Radiotherapy vs. Radiofrequency Ablation in the Treatment of Hepatocellular Carcinoma: A Meta-Analysis

**DOI:** 10.3389/fonc.2021.633417

**Published:** 2021-05-18

**Authors:** Zhaonan Li, Dechao Jiao, Xinwei Han

**Affiliations:** Department of Interventional Radiology, The First Affiliated Hospital of Zhengzhou University, Zhengzhou, China

**Keywords:** minimally invasive treatment, meta-analysis, hepatocellular carcinoma, stereotactic body radiotherapy, radiofrequency ablation

We were very interested in reading the article by Pan et al. (1), which included 10 studies involving 2,732 patients to compare the efficacy of stereotactic body radiotherapy (SBRT) and radiofrequency ablation (RFA) in the treatment of hepatocellular carcinoma (HCC). We appreciate the writing intention of this meta-analysis. However, there are several issues in the article, which are worthy of comment and adequate emphasis.

Firstly, local tumor recurrence (LTR) can be assessed only by ensuring that the tumor is completely ablated, that is to say, complete ablation of the tumor can be confirmed by imaging standards (2). Actually, for the two treatment methods currently discussed, only RFA can achieve the immediate posttreatment evaluation. It is worth noting that the imaging findings of HCC treated with SBRT changed gradually over time, and the weakening of the enhancement of HCC tends to precede the reduction in tumor size. As a matter of fact, the imaging findings of HCC after SBRT were not consistent with those of other focal therapies. At present, response evaluation criteria for solid tumors (RECIST) are placed on the basis of changes in tumor size, but the necrotic areas after SBRT are not considered in RECIST. Although the modified RECIST (mRECIST) and the European Association for the Study of Liver (EASL) criteria take into consideration live tumor within the lesion, the SBRT post-reaction for HCC has not been verified.

Secondly, despite the fact that the definition of LTR after RAF is very clear, the imaging evaluation of LTR after SBRT is particularly complicated and controversial. What included in the current assessment mainly are as follows: changes in the size of the target lesion, changes in internal enhancement characteristics, and assessment of necrosis by evaluating unenhanced areas within the tumor. Since the imaging manifestations of tumor imaging gradually change over time, the timing of imaging after SBRT is also important in evaluating treatment effects. Actually, after SBRT, the tumor is divided into three stages, including acute stage (<3 months), subacute stage (3–6 months), and chronic stage (3). In the acute stage and the subacute stage, the arterial phase hyperenhancement can sustain or subside in the portal phase, while a hyperenhancement at the delayed phase mainly occurs in the subacute phase. The fact that the reduction in enhancement of liver tumors treated with SBRT usually is ahead of the reduction in tumor size is really remarkable (4). Price et al. revealed that at each time point of 3, 6, 9, and 12 months, the estimated percentage of necrosis (assessed by non-enhancement within the lesion) is greater than the percentage of tumor shrinkage (5). Brook et al. reported that the weakening of tumor enhancement appeared very early (CT findings 15–45 days after SBRT) and persisted. In pathological evaluation, lesions with weakened enhancement but stable size may be severely necrotic (6). Therefore, when determining the initial response to SBRT, the size should support the changes in the enhancement of HCC. Due to the lack of imaging features of tumor recurrence after SBRT, the reliability of SBRT and RFA in the comparison of LTR is seriously weakened.

In addition, the term LTR refers to the emergence of new tumor foci at the ablative margin after local eradication of all tumor cells with ablation (2). However, there is considerable heterogeneity in the definition of LTR in the included SBRT studies, greatly influencing the feasibility of the conclusion. Wahl et al. defined LTR as a progression occurring at the planned target volume, which was constructed by expanding the clinical target volume by 5 mm radially and 8 mm craniocaudally (7). Shiozawa et al. simply clarified LTR as the recurrence of lesions after treatment (8), while Mohamed et al. did not clearly define local tumor recurrence in the literature (9). Furthermore, in a recent SBRT study involving 290 people, LTR was defined as a washout during the portal and delayed phases or increase in volume within the irradiated parenchyma (10). Therefore, this meta-analysis has a bias in the inclusion of LTR, and the necessity of avoiding using such data to complete the research ought not to be ignored.

In conclusion, the SBRT is an alternative option for the treatment of HCC. With the advent of high-precision image-guided radiotherapy (IGRT), SBRT could safely provide a sufficient radiation dose to tumors under the diaphragm or tumors close to important organs with the usage of various IGRT methods. In addition, IGRT with respiratory motion management (breath-hold techniques, 4-dimensional CT, or respiratory gating) also has the capacity to decrease toxicities and improve the therapeutic ratio. A growing amount of evidence suggests that both RFA and SBRT are effective local treatment options for inoperable HCC. Although these data are retrospective, SBRT appears to be a reasonable first-line treatment of inoperable, larger HCC. Ultimately, we appreciate the authors' efforts in exploration of the treatment with HCC. However, we sincerely suggest that appropriate modification would further confirm and greatly solidify the conclusions of the study.

## Author Contributions

XH: guarantor of the article. ZL, DJ, and XH: designed the study and wrote the paper. All authors approved the final version of the manuscript.

## Conflict of Interest

The authors declare that the research was conducted in the absence of any commercial or financial relationships that could be construed as a potential conflict of interest.

## Abbreviations

HCC: Hepatocellular carcinoma
LTR: Local tumor recurrence
RFA: Radiofrequency ablation
SRFA: Stereotactic radiofrequency ablation.
